# Global expression changes resulting from loss of telomeric DNA in fission yeast

**DOI:** 10.1186/gb-2004-6-1-r1

**Published:** 2004-12-15

**Authors:** Jeffrey G Mandell, Jürg Bähler, Thomas A Volpe, Robert A Martienssen, Thomas R Cech

**Affiliations:** 1 Department of Chemistry and Biochemistry and Howard Hughes Medical Institute, University of Colorado, Boulder, CO 80309-0215, USA; 2 The Wellcome Trust Sanger Institute, Cambridge, CB10 1SA, UK; 3 Cold Spring Harbor Laboratory, Cold Spring Harbor, NY 11724, USA

## Abstract

**Background:**

*Schizosaccharomyces pombe *cells lacking the catalytic subunit of telomerase (encoded by *trt1*^+^) lose telomeric DNA and enter crisis, but rare survivors arise with either circular or linear chromosomes. Survivors with linear chromosomes have normal growth rates and morphology, but those with circular chromosomes have growth defects and are enlarged. We report the global gene-expression response of *S. pombe *to loss of *trt1*^+^.

**Results:**

Survivors with linear chromosomes had expression profiles similar to cells with native telomeres, whereas survivors with circular chromosomes showed continued upregulation of core environmental stress response (CESR) genes. In addition, survivors with circular chromosomes had altered expression of 51 genes compared to survivors with linear chromosomes, providing an expression signature. *S. pombe *progressing through crisis displayed two waves of altered gene expression. One coincided with crisis and consisted of around 110 genes, 44% of which overlapped with the CESR. The second was synchronized with the emergence of survivors and consisted of a single class of open reading frames (ORFs) with homology both to RecQ helicases and to *dh *repeats at centromeres targeted for heterochromatin formation via an RNA interference (RNAi) mechanism. Accumulation of transcript from the ORF was found not only in *trt1*^- ^cells, but also in *dcr1*^- ^and *ago1*^- ^RNAi mutants, suggesting that RNAi may control its expression.

**Conclusions:**

These results demonstrate a correlation between a state of cellular stress, short telomeres and growth defects in cells with circular chromosomes. A putative new RecQ helicase was expressed as survivors emerged and appears to be transcriptionally regulated by RNAi, suggesting that this mechanism operates at telomeres.

## Abstract 2

Gene expression profiling of the response of *Schizosaccharomyces pombe *cells to loss of the catalytic subunit of telomerase (*trt1*^+^) identified two waves of altered gene expression and a continued up-regulation of Core Environmental stress Response (CESR) genes.

## Background

Telomeres are the nucleoprotein ends of linear eukaryotic chromosomes. In most organisms, telomeric DNA consists of a simple, repeated sequence with a G-rich strand running 5' to 3' towards the chromosome end, and terminates with a short, single-stranded 3' overhang (reviewed in [1,2]). The length of the duplex repeated region varies, from 20 base-pairs (bp) in hypotrichous ciliated protozoa to around 300 bp in yeast and several kilobases (kb) in mammalian cells. These DNA repeats recruit telomeric proteins to form the telosome, a structure that resists nucleolytic degradation and prevents chromosome ends from eliciting recombination and end-joining pathways for repairing double-strand DNA breaks [3].

Telomeres are also essential for the complete replication of chromosomes, because conventional DNA polymerases do not copy the extreme ends of linear DNA molecules. In the absence of a mechanism to compensate for this 'end-replication problem', progressive telomere shortening leads to replicative senescence, which in yeast is characterized by chromosome instability and low cell viability [4,5]. Replicative senescence in mammals is characterized by growth arrest and altered gene expression [6]. The end-replication problem is managed in most eukaryotes by the enzyme telomerase, which adds telomeric DNA sequences to the 3' end of chromosomes through the action of its catalytic subunit and RNA template (reviewed in [7]). DNA polymerase then forms duplex DNA by synthesizing the complementary C-rich strand of the telomere [8]. In fission yeast, the catalytic subunit of telomerase is encoded by the gene *trt1*^+ ^[9].

In some cases, cells can endure the loss of telomerase and give rise to a population of survivors. In the budding yeast *Saccharomyces cerevisiae*, survivors maintain long, heterogeneous telomeres on linear chromosomes using a *RAD52*-dependent homologous-recombination pathway [10]. Global gene-expression profiles of budding yeast lacking telomerase revealed the induction of a DNA damage response when telomeres were short and a sustained stress response in survivors [11]. Human alternative lengthening of telomeres (ALT) cells are cancerous cells lacking detectable telomerase activity that maintain long, heterogeneous telomeres using what is believed to be a strand invasion mechanism [12,13]. *S. pombe *cells without telomerase cease dividing after about 120 generations, and can give rise to a subpopulation of survivors [14]. Interestingly, these survivors have either circular chromosomes or linear chromosomes with long, heterogeneous amplified telomeres (presumably maintained through recombination) that resemble their budding yeast and human ALT-cell counterparts. While survivors with circular chromosomes arise more frequently, those with linear chromosomes grow faster [14].

Circular chromosomes in *S. pombe *are believed to form as a result of the genomic instability due to loss of telomeres, which normally prevent end-joining and suppress recombination. Interchromosomal fusions yield unstable dicentric chromosomes, while intrachromosomal fusions produce circular chromosomes. *S. pombe*, with only three chromosomes, is more likely than other organisms with larger numbers of chromosomes to successfully form exclusively intrachromosomal fusions [14,15]. *S. pombe *strains with circular chromosomes also result after concurrent deletion of *rad3*^+ ^and *tel1*^+^, two genes with sequence similarity to human *ATM *(ataxia telangiectasia mutated) [15].

Although *S. pombe *survivors with linear chromosomes grow remarkably well and have a morphology similar to wild-type cells, survivors with circular chromosomes display obvious growth defects such as slower growth rates and larger sizes [14]. Survivors with circular chromosomes presumably cope with impaired DNA segregation, and perhaps DNA breakage and rearrangement. We hypothesized that cells would show altered expression of genes necessary for coping with the loss of telomerase and concomitant changes in chromosome structure. In this study, we determined the *S. pombe *global gene-expression response to loss of *trt1*^+ ^to investigate changes in expression of genes during senescence, and to compare survivors with circular or linear chromosomes. We report that survivors with circular chromosomes maintain an extended stress response not observed in survivors with linear chromosomes. Furthermore, we present evidence for regulation of a telomeric gene by the RNAi machinery.

## Results

### Wild-type reference strains

**Figure 1:**
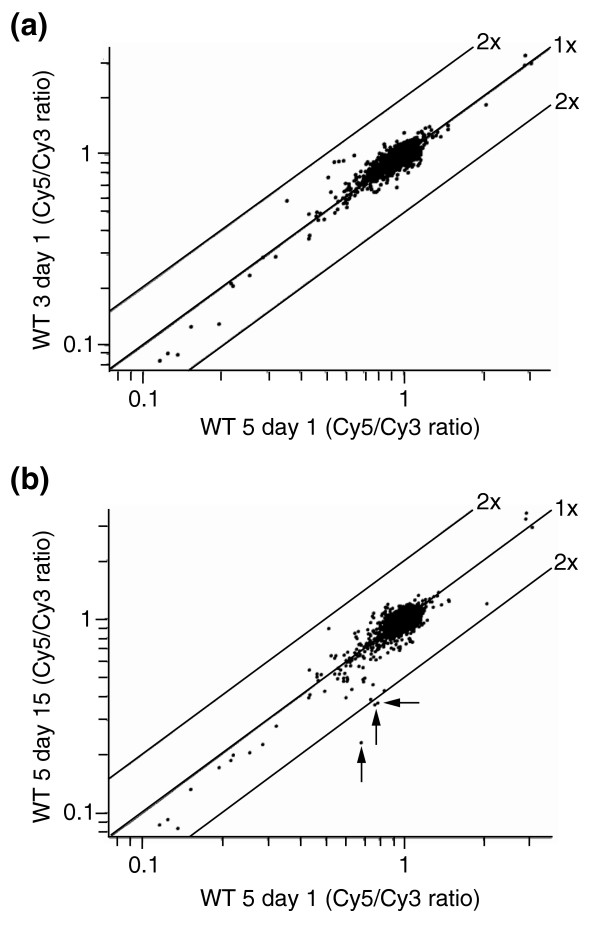
Stability of wild-type strain gene expression profiles. **(a) **Microarray expression data for two wild-type biological replicates, WT 3 and WT 5, on day 1 of the growth curve are plotted against each other. The expression data plotted are the normalized ratio of dyes Cy5- and Cy3-dCTP representing sample and reference pool, respectively. Lines showing limits of twofold change are drawn on both sides of the line of identity (identical values between datasets). The axes are log scale. Every gene for which there is data is shown (filled circles). All genes fall within the lines of twofold change. **(b) **As in (a), except WT 5 from day 1 of the growth curve is compared with WT 5 from day 15. Only three out of 5,050 genes, marked with arrows, changed expression by more than twofold. These genes are SPBC354.08c, encoding a hypothetical protein (2.15-fold); *atp8*^+^, F_0_-ATP synthase subunit 8 (2.15-fold); and *cox1*^+^, cytochrome *c *oxidase subunit I (2.98-fold).

Wild-type isogenic reference strains WT 3 and WT 5 were used to determine relative gene-expression changes in *trt1*^- ^samples. Before averaging the expression values from the two reference strains, the similarity of their expression profiles was assessed. The dye ratios measured by microarray for each strain were plotted against each other (Figure 1a). All genes had expression values that varied less than twofold between the two samples, indicating that the samples were highly similar. The wild-type values used in this paper are thus the average expression values of strains WT 3 and WT 5.

To learn whether changes in gene expression would result from subjecting cells to the continuous growth program for 15 days, gene-expression values from strain WT 5 on day 1 of the growth curve were compared with those of the same strain harvested on day 15 (Figure 1b). Only three genes (SPBC354.08c, *atp8*^+ ^and *cox1*^+^) changed their expression values by more than twofold, and they were only slightly greater; thus, the vast majority of genes do not have altered expression as a result of long-term growth in culture, provided that expression is measured while the cells are in early log phase (see Materials and methods). These three genes also had expression changes of more than twofold in one or more conditions measured for *trt1*^- ^cells, but given their variable expression in wild-type cells, these changes were most probably unrelated to the absence of telomerase.

### Watching cells pass through crisis and characterizing survivors

**Figure 2:**
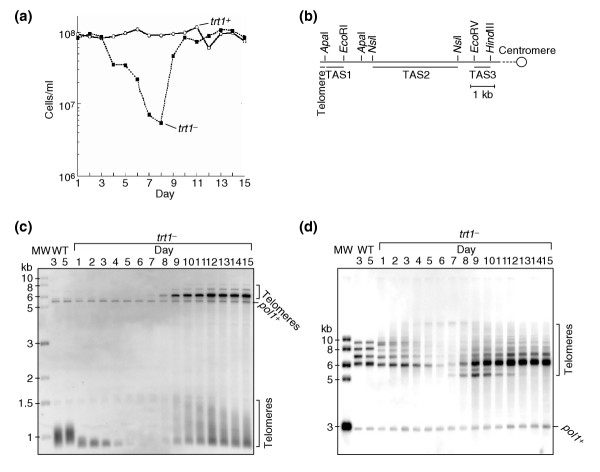
Senescence and emergence of survivors in *trt1*^- ^cells. **(a) **Growth curves. YES cultures (200 ml) were inoculated at 2.5 × 10^4 ^cells/ml with either *trt1*^+ ^or *trt1*^- ^cells. Cell density is shown for *trt1*^+ ^cells (open circles) and *trt1*^- ^cells (filled squares) at the end of each 24-h period, after which a new culture was inoculated at 2.5 × 10^4 ^cells/ml. When cells were counted on day 1, they had already undergone about 45 generations after germination. Note that when the culture density reached 3-5 × 10^6 ^cells/ml, a portion of the cells was harvested for microarray analysis and Southern hybridization. Cells appeared enlarged near day 8 and were morphologically normal by day 11. **(b) **Restriction-enzyme sites in the TAS of one chromosome arm cloned into the plasmid pNSU70 [58]. Locations of the probes used for Southern hybridization are indicated by the bottom bars. These probes hybridize to multiple chromosome arms because the TASs are found on the four arms of chromosomes I and II and, depending upon the strain background, on one or both arms of chromosome III. **(c) **Telomere length in wild-type and *trt1*^- ^strains from the growth curve. DNA (~15 μg) was digested with *Eco*RI, subjected to electrophoresis, transferred to a nylon membrane and probed with the ^32^P-labeled telomere fragment shown in (b) that was expected to report the state of the telomere end. As a loading control, a probe for the single-copy gene *pol1*^+ ^was included. Signals arising from the telomeres are labeled. **(d) **As in (c), but DNA was digested with *Hind*III and the blot probed with TAS2 and a fragment of *pol1*^+^. The TAS2 probe was expected to hybridize to sequences at least 2 kb, and up to 6 kb, from the telomere end.

**Figure 3:**
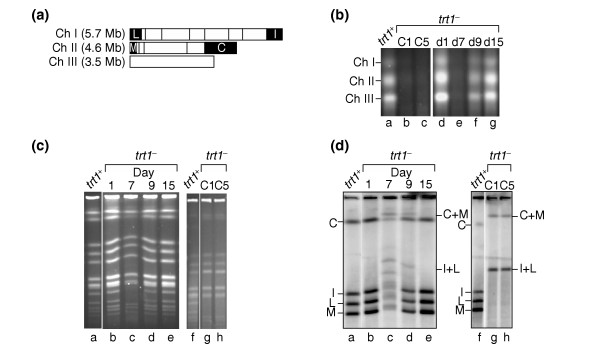
Chromosome structures of *trt1*^- ^survivors. **(a) **The 13 *Not*I restriction sites in *S. pombe *chromosomes I and II [65] are indicated by vertical lines. Chromosome III does not have a *Not*I site. Terminal fragments are labeled according to convention and highlighted in black. **(b) **Pulsed-field gel analysis of intact chromosomes visualized by staining with ethidium bromide. Lanes d-g correspond to days 1, 7, 9 and 15 of the growth curve, respectively. **(c) **Pulsed-field gel of *Not*I-digested chromosomes visualized with ethidium bromide. Days 1,7, 9 and 15 correspond to days of the growth curve. Lanes a and f were repositioned from the original gel image. **(d) **The gels from (c) were transferred to a nylon membrane and probed with a mixture of ^32^P-labeled probes to internal regions of the C, I, L and M fragments, identified in (a). The terminal fragments of linear chromosomes are labeled on the left, and fragments C+M and I+L resulting from circularized chromosomes are shown on the right.

Diploid *S. pombe *cells that were heterozygous for *trt1*^+ ^and able to maintain full-length telomeres were sporulated, and the resulting *trt1*^+ ^and *trt1*^- ^cells propagated through a 15-day growth curve (Figure 2a). Cells lacking telomerase gave rise to survivors after day 8 concomitant with heterogeneous amplified telomeric repeats and telomere-associated sequence (TAS) (Figures 2b-d), indicative of linear chromosomes [14]. By day 15, the culture was dominated by faster-growing cells with linear chromosomes. The linear structure of these chromosomes was confirmed by their ability to enter a pulsed-field gel (Figure 3b, lane g), and the existence of terminal chromosome fragments C, I, L and M after digestion of chromosomes with *Not*I (Figure 3a-d, lane e) [14,15]. Cells passing through crisis (days 7 and 9) also had weak hybridization signals for the C+M and I+L fragments (Figure 3d, lanes c-d), suggesting a mix of cells with either linear or circular chromosomes, or perhaps cells containing both linear and circular chromosomes. The inability to detect intact chromosomal DNA at day 7 (Figure 3b, lane e) may have resulted from the presence of cells with circularized chromosomes (Figure 3d, lane c) that do not enter pulsed-field gels.

Strains C1 and C5 had circular chromosomes as evidenced by lack of telomeric repeats (data not shown), lack of TAS2 sequence (data not shown), the inability of chromosomes to enter a pulsed-field gel (Figure 3b, lanes b-c), the lack of terminal chromosome fragments C, I, L and M (Figures 3c,d, lanes g-h) [14,15], and hybridization signals to fragments C+M and I+L (Figure 3d, lanes g-h).

### Two waves of expression are observed in the growth curve

**Figure 4:**
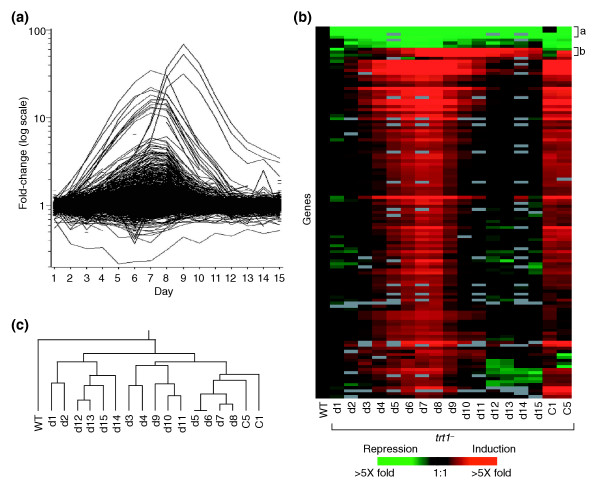
Gene-expression profiles of cells experiencing senescence and survivors. **(a) **Graph of expression for all genes showing fold-change relative to wild type for each day of the growth curve. Each gene is represented as a line with discontinuities resulting from missing data. For clarity, three genes (missing from the genome, see text) with expression reduced tenfold or more are not shown: *trt1*^+^, SPAC2E1P3.04 and SPAC2E1P3.05c. **(b) **Hierarchical clustering of the 123 genes whose expression changed by twofold or more relative to wild-type in two or more days of the growth curve (see text for details). Samples d1-d15 are days of the growth curve. Each column represents expression of all 123 genes for a unique condition. Each row represents the expression pattern of a single gene throughout all conditions. Genes shown in red had upregulated expression and those in green had downregulated expression. Values of fold-change less than 1.2 are in black, and gray areas indicate missing data. Brackets labeled with letters a-b along the right-hand side denote sets of genes with similar expression patterns for one or more conditions. Band 'a' consists of genes with downregulated expression: SPAC2E1P3.05c, SPAC2E1P3.04, *trt1*^+ ^and SPBC359.02; and band 'b' represents the second wave of gene expression in the growth curve. The wild-type sample was an average of biological replicates WT 3 and WT 5. **(c) **Dendrogram of the experimental conditions and strains shown in (b). Experiments were hierarchically clustered on the basis of the similarity of expression ratios of the 123 genes shown in (b).

Two waves of altered gene expression were seen during the growth curve (Figure 4a), the first with a peak at day 7, consisting of around 110 genes with expression upregulated twofold or more, and the second with a peak at day 9, consisting of three microarray signals that appear to represent a single ORF (see below) (Figure 4a). The peak of the first wave (day 7) was nearly coincident with crisis in the cell population (day 8) (Figure 2a) and the time when telomeres were shortest (near day 7) (Figure 2c,d). The second peak of gene expression at day 9 was coincident with the emergence of survivors (Figure 2a-d).

The vast majority of expression changes involved upregulation, and only seven genes had downregulated expression of twofold or greater on two or more days of the growth curve. Notably, there were three cases of reduction in expression greater than tenfold: *trt1*^+ ^(intentionally knocked out), SPAC2E1P3.04 (a predicted copper amine oxidase) and SPAC2E1P3.05c (unknown function). Hybridizations of genomic DNA to microarrays (data not shown) revealed that genes SPAC2E1P3.04 and SPAC2E1P3.05c were deleted from the genome in all strains except WT 3, WT 5 and C1. Interestingly, these two genes are within about 4 kb of transposable element SPAC167.08 (Tf2-2), suggesting a hotspot for DNA excision. In no case was gene amplification detected by genomic hybridization (data not shown), so the observed increases in expression were most probably due to transcriptional or post-transcriptional regulation, as opposed to changes in gene copy number.

### Gene-expression changes in *trt1*^- ^cells

Because a relatively large number of *trt1*^- ^strains were studied, the identification of genes with consistently altered expression was facilitated by selecting those genes with expression changes of twofold or more in two or more days of the growth curve or, alternatively, in both strains C1 and C5. This criterion was met by 123 genes, of which 54 (44%) overlapped between the growth curve and survivors with circularized chromosomes. In addition, of the 67 genes that had their expression changed twofold or more exclusively in the growth curve, many displayed altered expression just below the cutoff in survivors with circularized chromosomes. Two genes - SPBC1683.06c (a predicted uridine ribohydrolase) and SPBC1198.01 (a predicted formaldehyde dehydrogenase) - had expression changes of twofold or more in both strains C1 and C5, but no significant changes during the growth curve. As a measure of confidence, 84 of the 123 genes (approximately 68%) met a more stringent criterion requiring a gene to change its expression in three or more of the 17 conditions. Additional confidence that expression changes scored as significant were not false positives came from the remarkably continuous manner in which gene expression changed throughout the growth curve (Figure 4a).

The 123 genes with altered expression encompass a broad range of functions, but were especially enriched in genes associated with energy production and carbohydrate metabolism (Table 1). There were seven pseudogenes and 29 predicted genes that did not have assigned functions at the time of writing. For nearly all the gene-type categories, there was a larger number of genes with altered expression in the growth curve than in the survivors with circular chromosomes (Table 1). This difference may be attributable to the fact that cells in the growth curve were experiencing crisis whereas strains C1 and C5 were survivors, presumably with established mechanisms to cope with the absence of or the loss of telomeres.

The telomerase-deletion response had a large overlap with genes that changed expression in response to environmental stresses. Fission yeast stress-response genes can be separated into a CESR, in which genes changed expression in all or most of the stresses studied (oxidative stress, heavy metals, heat shock, osmotic stress and DNA damage), and into more specific stress responses [16]. Of the 123 genes with altered expression in *trt1*^- ^cells, 48 (about 39%) also had upregulated expression among a conservative list of CESR genes (*P *~ 10^-77^) [16], and two genes had downregulated expression in the CESR and in this study. Of the 110 genes with expression upregulated twofold or more on day 7 of the growth curve, 44% overlapped with the CESR. Comparison with a less conservative list of CESR genes [16] suggested that 54% of the 123 genes with altered expression in *trt1*^- ^cells had overlap with the CESR (*P *~ 10^-81^). With respect to specific stress responses [16], there were 17/123 genes in common with the oxidative stress response (*P *~ 10^-32^), and 11/123 genes in common with the heat stress response (*P *~ 10^-24^). The stress response study found that the DNA damage response and the oxidative stress response have substantial overlap [16]. Therefore, the genes with altered expression in this study that overlap with the oxidative-stress response may represent a DNA damage response to short telomeres.

### Chromosome structure and gene expression

Comparisons of all the gene-expression profiles in this study revealed striking differences between the profiles of survivors with linear chromosomes versus those with circular chromosomes. Survivors with linear chromosomes (days 12-15 of the growth curve) had gene-expression patterns similar to those of cells with native telomeres in the first two days of the growth curve. To illustrate, by day 12 of the growth curve, the gene-expression profiles of survivors became relatively constant and remained so through day 15. The profiles of days 12-15 appear most similar to days 1 and 2 of the growth curve, immediately after cells lost telomerase and were experiencing shortening telomeres (Figure 4b). This observation was confirmed by hierarchical clustering (Figure 4c). Conversely, survivors with circular chromosomes had gene-expression profiles that most resembled those of cells in crisis during days 5-8 of the growth curve (Figure 4b,c).

### Sustained stress response in survivors with circular chromosomes

**Figure 5:**
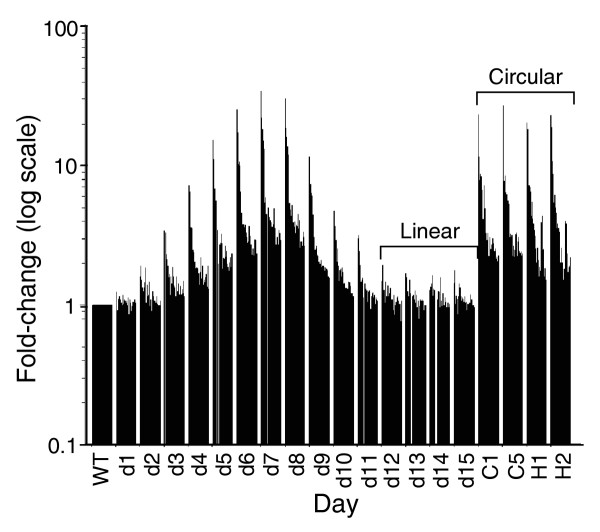
Expression signatures of cells with circular chromosomes. For each condition, the 51 genes from Table 2 that had expression changes of twofold or more in both strains C1 and C5, but not in survivors with linear chromosomes, are graphed in clusters of vertical bars. The height of each bar represents fold-change in expression relative to wild type. Survivors with linear or circular chromosomes are labeled. Strains H1 and H2 have circular chromosomes as evidenced by their inability to enter into a pulsed-field gel (data not shown). Strains H1 and H2 were not used to derive the expression signature and are shown as an independent verification of it.

There were 54 genes with clearly altered expression (twofold or more) mainly during crisis in the growth curve that also had altered expression in the survivors with circular chromosomes (Table 2, Figure 5). The expression of all but three of these 54 genes was not altered in survivors with linear chromosomes (growth curve days 12-15) (Table 2). Of the 54 genes, 30 (56%) overlapped with the conservative list of CESR genes (*P *~ 10^-46^), and eight genes (15%) overlapped with the oxidative stress response (*P *~ 10^-14^). There were 8/54 genes (15%) that overlapped with the heat stress response (*P *~ 10^-17^). Because of the extensive overlap of the 54 genes with the CESR, we conclude that survivors with circular chromosomes had a sustained stress response.

Of the 54 genes, 51 represent a gene-expression signature that differentiates survivors with circular chromosomes from those with linear chromosomes. As an independent test of whether these 51 genes can serve as a signature for cells with circularized chromosomes, two additional cultures (strains H1 and H2, see Materials and methods) with circularized chromosomes were grown and analyzed by microarray. Both strains clearly displayed altered expression of the 51 genes whereas survivors with linear chromosomes did not (Figure 5), thus validating this gene signature.

### No altered expression of genes encoding recombination and telomere factors

One feature of microarray studies is that genes not previously recognized to be under the control of a common regulator can often be associated by similar expression patterns [17]. On the basis of this hypothesis, a list of genes known to be involved in telomere maintenance and recombination was inspected. However, the expression patterns of all these genes were not substantially changed throughout the course of the study (data not shown). Genes investigated included *pku70*^+ ^and *lig4*^+^, which encode components of the non-homologous end-joining pathway [18]; *taz1*^+ ^[19] and *pot1*^+ ^[20] encoding telomere DNA-binding proteins; telomerase component *est1*^+ ^[21]; homologous recombination-related genes *rad22*^+ ^[22], *rhp54*^+ ^[23], *rad32*^+ ^[24] and *rhp51*^+ ^[25]; RecQ helicase gene *rqh1*^+ ^[26]; silencing component *clr4*^+ ^[27]; and telomere maintenance components *pof3*^+ ^[28] and *rad3*^+ ^[15]. Interestingly, even though *pof3*^+ ^and *clr4*^+ ^expression did not change, the genes with altered expression in this study had a statistically significant overlap with the lists of genes with induced expression in *pof3 *mutants (*P *< 10^-45^) [28] and *clr4 *mutants (*P *< 10^-45^) [29]; a significant correlation was also observed with genes that changed expression in the RNA interference (RNAi)-machinery mutants *dcr1*^+^, *ago1*^+ ^and *rdp1*^+ ^(*P *~ 10^-22^) [29]. These genes with altered expression may act in common pathways downstream of *trt1*^+^, *clr4*^+^, *pof3*^+ ^and the RNAi machinery.

### A second wave of expression represents sub-telomeric ORF with homology to RecQ helicases and *dh *repeats

The second wave of gene-expression changes during the growth curve (Figure 4a) consisted of three microarray signals: SPAC212.11 (largest magnitude), SPAC212.06 (second largest magnitude) and the reverse transcript of centromeric *dh *repeats [30]. Inspection of the sequences revealed that the microarray signals from SPAC212.06 and centromeric *dh *repeats most probably resulted from cross-hybridization with the SPAC212.11 transcript (see Materials and methods).

**Figure 6:**
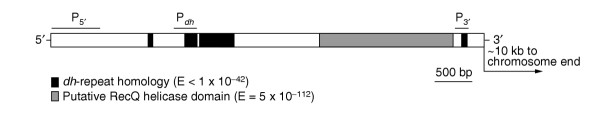
Homology of the putative helicase with RecQ helicases and *dh *repeats. The 5.6 kb sequence of SPAC212.11 is represented as a rectangle. Horizontal lines above the gene indicate the regions spanned by primers used in this study. P_3' _was the fragment of SPAC212.11 on the microarray (180 bases), and P_5' _was used in Southern hybridizations (642 bases). Region P_*dh *_was amplified in RT-PCR experiments (Figure 7) to detect *dh *repeat forward and reverse strands. Solid black rectangles are regions of homology with *dh *repeats found at centromeres and in the *K *region of the mating-type locus. The predicted amino-acid sequence of the region marked with cross-hatching has homology with the RecQ helicase family. The BLAST expect (E) value is shown, with the exception that the approximately 70 bp region of homology to *dh *repeats 3' of the putative RecQ helicase domain has an E value of 2 × 10^-8^.

A BLAST search of the SPAC212.11 predicted protein sequence found that the ORF has the most similarity to RecQ DNA helicases of superfamily II (Figure 6) (reviewed in [31]). We report a role for the helicase in cells passing through crisis in a separate study (J.G.M., K.J. Goodrich, J.B. and T.R.C., unpublished work) and investigate its transcriptional regulation here.

SPAC212.11 is the last sequenced ORF on the left arm of chromosome I. The sub-telomeric regions of chromosomes I and II have significant similarity [32]. A BLAST search performed with the SPAC212.11 DNA sequence (5.6 kb) revealed a paralog, SPBCPT2R1.08c (6.3 kb), located on the right arm of chromosome II (the microarray had no probe for SPBCPT2R1.08c), and partial homology on the right arm of chromosome I. The annotated sequence of SPBCPT2R1.08c includes the entirety of the SPAC212.11 sequence with only a single base change. The SPAC212.11 sequence does not contain a stop codon because the ORF is located at the end of the sequencing contig, which ended before a stop codon was reached. Comparison with the annotated SPBCPT2R1.08c sequence suggests that SPAC212.11 has an additional 95 bp before the stop codon.

Both SPBCPT2R1.08c and SPAC212.11 are the last predicted genes on their respective sub-telomeric sequencing contigs. Analysis of contig pT2R1 revealed that the 3' end of SPBCPT2R1.08c is approximately 2.8 kb upstream from the start of TAS3 (Figure 2b). Since TAS3 is around 7 kb from the chromosome end, the 3' end of SPBCPT2R1.08c is approximately 10 kb from the telomeric repeats.

It is not known which of the paralogs contributed to the SPAC212.11 microarray signal. For the sake of simplicity, further references in the text to 'the putative helicase' are meant to include SPAC212.11, SPBCPT2R1.08c and any paralogs, collectively.

The nucleotide BLAST search performed with the SPAC212.11 sequence also revealed that the ORF contains regions of homology to *dh *repeats (Figure 6), which are targeted for heterochromatin formation via an RNAi-mediated mechanism in *S. pombe *[33,34]. These repeats are typically located at centromeres and the *K *region of the mating-type locus [30,33,35-37].

### RNAi machinery implicated in controlling expression of the putative helicase

Centromeric repeats, previously thought to be transcriptionally silent, are transcribed in both the forward and reverse directions, leading to formation of double-stranded RNA (dsRNA). However, these transcripts do not accumulate in wild-type cells. Reverse-strand centromeric transcripts are synthesized and rapidly processed by the RNAi machinery, while forward-strand synthesis is silenced transcriptionally. RNA-dependent RNA polymerase (Rdp1) associates with centromeric repeat DNA and may use siRNAs corresponding to centromeric transcripts [38] to prime forward transcription from reverse-strand templates, thus resulting in dsRNA formation and maintenance of the heterochromatic state. In the RNAi mutants *dcr1*^-^, *ago1*^- ^and *rdp1*^-^, centromeric silencing is abolished and accumulation of both forward and reverse centromeric transcripts is observed [33].

**Figure 7:**
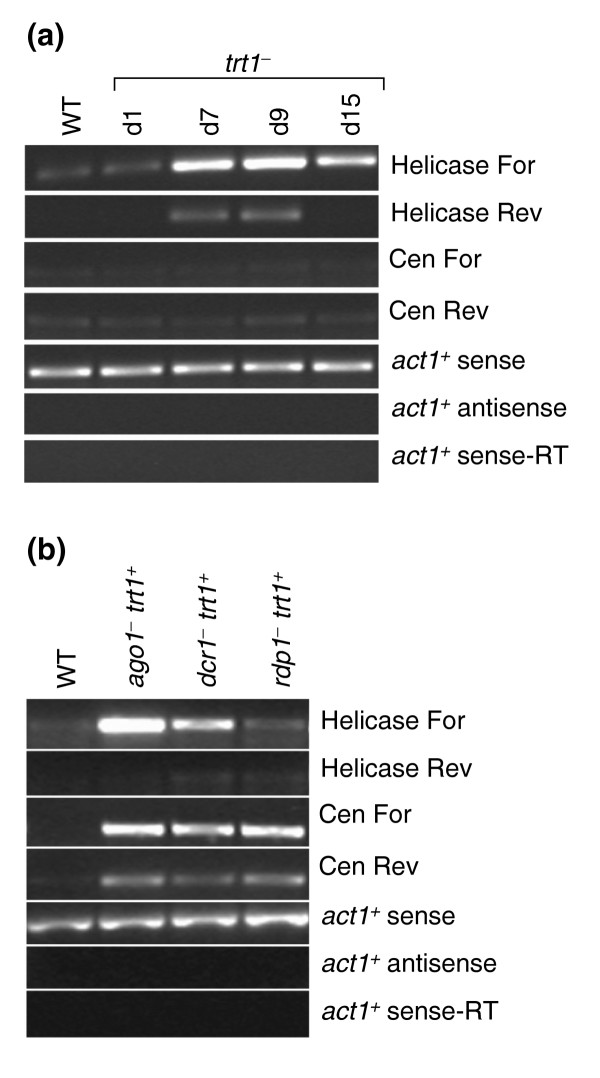
Expression of *dh *repeats at the sub-telomere. **(a) **Expression of sub-telomeric *dh *repeats in *trt1*^- ^mutants. Strand-specific RT-PCR using primers spanning the region of *dh *repeats in the putative helicase (P_*dh *_in Figure 6) was used to detect the expression of both forward (For) and reverse (Rev) transcripts. We define the forward transcript to be homologous to the DNA strand running towards the chromosome end in the 5' to 3' direction (this is also the strand with the longest ORF). Strand-specific control reactions were also performed using primers specific for centromeric (Cen) *dh *repeats [33], as well as *act1*^+ ^sense and *act1*^+ ^antisense transcripts (a control lacking reverse transcriptase is labeled -RT). Strains WT 5 and days 1, 7, 9 and 15 of the growth curve are shown. **(b) **Expression of sub-telomeric *dh *repeats in RNAi mutants. RNA was isolated from *trt1*^+ ^RNAi mutant strains *ago1*^-^, *dcr1*^- ^and *rdp1*^- ^[33], and subjected to strand-specific RT-PCR using the same primers described in (a). A different wild-type strain from that in (a) was used.

Microarray, northern blot and reverse transcription (RT)-PCR analysis indicated that the putative helicase gene was robustly expressed in cells emerging from crisis, but was weakly (or not at all) expressed in wild-type cells, strains C1 and C5 and survivors with linear chromosomes (Figures 4a,b, 7a, and data not shown). As the putative helicase transcript was not detectable by northern blot in wild-type cells (data not shown), we hypothesized that this ORF could be silenced by its *dh *repeats, but that this silencing may have been disrupted in *trt1*^- ^cells as a result of genomic instability. Arguing against this hypothesis, however, Southern analysis with probe P_5' _(Figure 6), which is specific for the helicase, did not reveal any DNA rearrangements during crisis close to the helicase that might have contributed to loss of silencing (data not shown). Nevertheless, the loss of silencing observed might lead to expression of both strands of the putative helicase, as was found for centromeric *dh *repeats in RNAi mutants.

To test for the presence of both strands, strand-specific RT-PCR was used with primers spanning the *dh *repeats of the putative helicase (region P_*dh *_in Figure 6). The forward strand was expressed at levels higher than in wild type in cells from days 7, 9 and 15 of the growth curve. These results were consistent with microarray analysis that detected the 3' end of the forward transcript (Figure 7a). The reverse strand was weakly detectable in cells from days 7 and 9 of the growth curve (Figure 7a).

dsRNA arising from the repeats presumably could have formed on days 7 and 9 of the growth curve, but why such RNA was not all processed by the RNAi machinery is not clear. On days 7 and 9 of the growth curve, the RNAi machinery was not apparently affected by the mutation of telomerase as centromeric *dh *repeat transcripts were not detected by RT-PCR (Figure 7a).

We next hypothesized that if the RNAi machinery were involved in transcriptional silencing of the putative helicase in wild-type cells, transcript should accumulate in mutant RNAi strains. Strikingly, both *ago1*^- ^and *dcr1*^- ^strains displayed significant accumulation of the forward transcript of the putative helicase, and the *rdp1*^- ^strain showed slightly increased accumulation with respect to wild-type (Figure 7b). The reverse strand did not accumulate in these three strains. Thus, transcriptional silencing of the putative helicase appeared to be relieved in RNAi mutants, implicating RNAi in the control of expression of this ORF.

## Discussion

### Correlation of chromosome structure and gene expression

The genome-wide survey of expressed genes in this study provided an opportunity to investigate the cellular response to loss of the gene for the telomerase catalytic subunit Trt1. A major finding was the tight correlation between the structures of chromosomes in survivors and gene expression profiles. Survivors with linear chromosomes had expression profiles remarkably similar to cells with canonical - yet shortened - telomeres, whereas cells with circular chromosomes maintained the upregulated expression of a significant number of genes that also had upregulated expression during senescence.

The stress response in survivors with circular chromosomes had significant overlaps with the *S. pombe *CESR and with the heat and oxidative stress responses. The CESR consists of genes that had upregulated expression in all or most responses to oxidative stress, heavy metal stress, heat shock, osmotic stress and DNA damage [16]. The stress response may persist in survivors with circularized chromosomes because of impaired DNA segregation and DNA breakage and rearrangement. Indeed, compared with wild-type cells, survivors with circular chromosomes are larger and have slower growth rates, indicating that functions related to cell division are impaired [14].

Telomeric repeats contribute to recruiting the molecular components collectively involved in the protective capping of chromosome ends [20,39,40]. These repeats are maintained in the absence of telomerase in cells from diverse organisms that normally use telomerase (reviewed in [3]). Interestingly, the survivors with linear chromosomes abated their stress response concomitant with the appearance of amplified telomeric and TAS repeats as rare survivors took over the population, suggesting that the repeats helped to ameliorate the stress response.

Neither cells in the growth curve that experienced shortened telomeres nor survivors with long telomeres displayed upregulation of telomeric gene expression, supporting the notion that telomeric length changes alone do not affect gene expression in *S. pombe *[19]. In addition, in survivors with circular chromosomes, only eight microarray signals, corresponding to as few as two genes (due to cross-hybridization) near former telomeres had altered expression, although such changes might have been expected as a result of the large alterations in chromosome structure at these sites.

### Comparison with the budding yeast response to loss of telomerase

As in fission yeast, genes with changed expression in the budding yeast response to loss of telomerase had significant overlaps with genes whose expression was altered by environmental stresses such as heat shock, osmotic shock, dithiothreitol (DTT)**, **nitrogen starvation and peroxide ([11,41] see also [42]). A difference in the stress responses between the two yeasts was that in budding yeast a large but specific subset of the environmental stress-response genes persisted in survivors with linear chromosomes four days after crisis, whereas in fission yeast survivors with linear chromosomes, the stress response mostly abated by the fourth day after crisis (Figure 4b, day 12). The different yeast responses may be due to a fission yeast telomere structure that was not as strongly recognized as aberrant, perhaps mitigating a DNA-damage response. It is also possible that had budding yeast survivors been followed longer, providing a period for adaptation, the stress response would have subsided.

In fission yeast, the expression of a number of mitochondrial ATP synthase genes was upregulated (Table 1) with orthologs similarly induced in budding yeast. In both cases, the changes did not overlap with the DNA-damage responses of the yeasts, further supporting a link between short telomeres and alterations in the metabolic program suggested by Nautiyal *et al*. [11].

### Significance of putative RecQ helicase

RecQ helicases have recently been implicated in telomerase-independent telomere maintenance in both *S. cerevisiae *and human ALT cells. BLM and WRN, human RecQ helicases associated with cancer and disease [31], have both been shown to associate with duplex telomere repeat binding protein TRF2 *in vivo*, and BLM co-localizes to telomeric foci exclusively in ALT cells [43-45]. The *S. cerevisiae *ortholog of human WRN and BLM, Sgs1, was also shown to be required for telomere elongation of type II survivors in the absence of telomerase [46-48]. The long, heterogeneous telomeres of *S. pombe *survivors with linear chromosomes are similar to those of *S. cerevisiae *survivors and human ALT cells, suggesting a role for RecQ helicases in fission yeast telomerase-independent telomere maintenance.

### *dh *repeats and RNAi at the telomere

This is the first report to our knowledge of naturally occurring *dh *repeats outside of the centromeric and mating-type regions in fission yeast. We have presented several results that suggest that sub-telomeric *dh *repeats promote heterochromatin formation at the helicase locus. First, transcript from this ORF was only weakly expressed in wild-type cells as determined by RT-PCR (Figure 7a) (and was not detectable at all by northern hybridization, data not shown), consistent with transcriptional regulation of this ORF by heterochromatin. Second, expression of the putative helicase was robust in *ago1*^- ^and *dcr1*^- ^mutants, which would be expected if RNAi has a role in transcriptionally silencing this ORF. In *trt1*^- ^mutants experiencing genomic instability, we detected both forward and reverse transcripts of sub-telomeric *dh *repeats (Figure 7a). The presence of these complementary transcripts suggests the existence of dsRNA that had not been processed by the RNAi machinery, consistent with a lack of silencing at this locus. Intriguingly, after maximal expression of both strands on day 9 of the growth curve, subsequent downregulation was observed by day 15 (Figure 7a), consistent with restoration of silencing.

While the finding of homology with *dh *repeats at the sub-telomere was unexpected, *dh *repeats have been shown to function in silencing at sites outside of centromeres and the mating-type locus. Reporter genes fused to centromeric repeat fragments as short as 580 bp were silenced when integrated at ectopic locations in the genome [49,50] and this silencing required the RNAi machinery [51,52]. The longest (nearly continuous) stretch of sequence with homology to *dh *repeats found in the helicase ORF was about 600 bp (Figure 6), presumably long enough to promote heterochromatin formation. In addition, RNAi-mediated silencing triggered by both a synthetic hairpin RNA and transposon long terminal repeats have been shown to induce heterochromatin formation away from centromeres and the mating-type locus [53].

In a separate study, telomeric silencing of a reporter gene and binding of Swi6 at the telomere were not affected in *dcr1*^-^, *ago1*^- ^and *rdp1*^- ^mutants [54]. The lack of an observed effect may have been due to the ability of telomeric repeats to recruit silencing factors. Indeed, telomeric heterochromatin is largely promoted by telomeric repeats. However, the study by Hall and co-workers [54] did report defective mitotic and meiotic telomere clustering in RNAi mutants, supporting a role for RNAi at telomeres.

Given the correlation between disruption of telomeric heterochromatin and expression of the helicase ORF, events other than telomere erosion that disrupt heterochromatin might also induce helicase expression.

## Materials and methods

### Strain construction

The *trt1*^+ ^and *trt1*^- ^cells used in this study were generated by sporulating *S. pombe *diploid strain G4 (*h*^-^/*h*^+ ^*ade6-M210*/*ade6-M216 trt1*^+^/*trt1*^-^) on ME plates [18]. The parent diploid strain was made heterozygous for *trt1*^+ ^by using a standard two-step integration procedure [55] with a linearized plasmid containing about 1 kb each of the 5' and 3' flanking regions of the *trt1*^+ ^ORF separated by *HSV1-tk *and *KanMX4 *[56]. The plasmid was linearized in the middle of the 3' flanking region with *Fse*I and transformed using the lithium acetate method [57] into a diploid strain created by crossing PP68 (*h*^- ^*ade6-M210*) and PP69 (*h*^+ ^*ade6-M216*). Cells were re-streaked twice on yeast extract low adenine (YEA) + geneticin plates [18] to select for stable genomic integrants, which were subsequently confirmed by Southern hybridization to a uniquely sized *Eco*RI restriction fragment 3' of *trt1*^+ ^which was present only in integrants. Cells were then plated on YEA + 50 μM 5-fluorodeoxyuridine (5-FUdR) plates to select for those that had excised *HSV1-tk*, KanMX4 and the *Xba*I-*Xho*I fragment (around 5 kb) of *trt1*^+ ^from their genomes. Random surviving colonies were screened for heterozygous diploids by Southern hybridization to the 3' region of the *trt1*^+ ^*Kpn*I restriction fragment. The heterozygous state was evidenced by hybridization signals to both full-length *trt1*^+ ^and a shortened, nonfunctional version. Loss of markers was confirmed by lack of a Southern hybridization signal to *HSV1-tk*, and by lack of growth on YEA + geneticin plates.

### Selection of strains

After germination of G4 and growth of spores at 32°C for three days on YEA plates [18], plates were stored at 4°C while the genotypes of random colonies were determined. A portion of single colonies was used for crossing and visual inspection to identify those that had an *h*^- ^*ade6-M210 *genotype, which were further screened by Southern hybridization for the presence or absence of *trt1*^+ ^(performed as described in 'Strain construction' below). Colonies were subsequently used as described in 'Growth curve', or alternatively used to create strains C1, C5, H1 and H2.

Strains C1, C5, H1 and H2 were created from four separate *trt1*^- ^colonies that were each successively re-streaked on YEA plates 15 times (with growth for 2 to 3 days at 32°C between re-streaks), to permit colonies to form without competition from faster-growing survivors with linear chromosomes. During this time cells were presumed to senesce and give rise to survivors. After the last re-streak, a single colony from each strain was randomly selected and used to prepare freeze stocks.

### Growth curve

Three strains were grown: two wild-type isolates (*h*^- ^*ade6-M210 trt1*^+^) designated WT 3 and WT 5, and a single mutant isolate (*h*^- ^*ade6-M210 trt1*^-^) designated as 'GC Day X', where X represents the day of the growth curve that cells were collected. Single colonies were used to inoculate 5-ml starter cultures in yeast extract full supplements (YES) medium [18] and grown for 24 h with shaking at 32°C. Cells were counted and used to inoculate 200-ml YES cultures in 500-ml Erlenmeyer flasks at 2.5 × 10^4 ^cells/ml, and were grown in an incubator (Innova 4430, New Brunswick Scientific) with continuous shaking at 200 rpm at 32°C. Cell density was monitored by periodic counting, and a portion of the cells was harvested for microarray analysis and Southern hybridization when the density reached 3-5 × 10^6 ^cells/ml (early log phase). Cells harvested at this point were referred to as day 1 of the growth curve. The unharvested cells were permitted to continue growing until 24 h from the time of inoculation, at which time cells were counted and used to inoculate a fresh 200-ml YES culture at 2.5 × 10^4 ^cells/ml, and the process repeated for 15 days. To harvest cells for microarray analysis, a volume of culture containing approximately 1.6 × 10^8 ^cells was gently centrifuged at room temperature (2,000 rpm for 2 min), the supernatant removed, and the cell pellet snap-frozen in liquid N_2_. For Southern hybridization, approximately 2 × 10^8 ^cells were collected by centrifugation, washed twice in H_2_O, and snap-frozen in liquid N_2_. A portion of cells for pulsed-field gel analysis was also collected in the same manner as for Southern hybridization at the end of each 24 h period. *trt1*^- ^cells were collected daily, WT 3 and WT 5 on day 1, and WT 5 on day 15.

### Growth and collection of strains C1, C5, H1 and H2

Cells were streaked onto YEA plates from freeze stocks, grown for 3 days at 32°C, and single colonies used to inoculate 5-ml starter cultures in YES medium. After 24 h, cells were counted and 200-ml YES cultures were inoculated at 2.5 × 10^4 ^cells/ml, and cultures grown with constant shaking at 200 rpm at 32°C. When the cell density reached around 3 × 10^6 ^cells/ml (early log phase), cells were collected as described in 'Growth curve' for microarray analysis, Southern hybridization and pulsed-field gel electrophoresis. Strains H1 and H2 are *trt1*^- ^isolates with circular chromosomes, as evidenced by pulsed-field gel electrophoresis (data not shown)

### Genomic DNA preparation and Southern hybridization

DNA from approximately 2 × 10^8 ^*S. pombe *cells was prepared as described [18]. After digestion with either *Eco*RI or *Hind*III, the DNA was subjected to electrophoresis on a 1% agarose gel in 1 × TBE (90 mM Tris, 90 mM borate, 2 mM EDTA pH 8.3). DNA was denatured by sodium hydroxide treatment and transferred to a nylon membrane (Hybond-N+ membrane, Amersham) by capillary transfer in 10 × SSC (1.5 M NaCl, 0.15 M sodium citrate). DNA was immobilized on the membrane by irradiation with 120 mJ/cm^2 ^at 254 nm in a UVStratalinker1800 (Stratagene). For molecular weight markers, the 1 kb DNA ladder (New England Biolabs) was labeled by filling in 5' overhangs with [α-^32^P]dATP using DNA polymerase I Klenow fragment. Probes for *pol1*^+^, *act1*^+^, the putative helicase and the C, I, L and M chromosome fragments were generated by PCR amplification from a genomic DNA template and were gel purified. Probes were labeled by random-primed transcription of PCR products with the use of [α-^32^P]dCTP and High Prime Mix (Boehringer Mannheim). Probes specific for the telomeric and telomere-associated sequences were created with the use of gel-purified fragments of pNSU70 [58].

### Pulsed-field gel electrophoresis

Cells (approximately 1 × 10^8^) were collected as described above. Plug preparation, chromosome digestion and electrophoresis were performed exactly as described [18]. DNA was visualized by staining with ethidium bromide (1 μg/ml) for 30 min. The gel was then irradiated with 120 mJ/cm^2 ^at 254 nm in a UVStratalinker1800 to nick the DNA, treated with HCl, NaOH and neutralization buffer, and processed as described in 'Southern hybridization'.

### RT-PCR

RNA was prepared as for microarray analysis and used for RT-PCR (OneStep RT-PCR kit, Qiagen). First-strand cDNA synthesis was performed using primers complementary to either the forward or reverse strands. Both primers were present in subsequent cycles of PCR amplification after heat inactivation of reverse transcriptase at 95°C for 15 min. The control reaction lacking reverse transcriptase (*act1*^+ ^sense, -RT) was not subjected to first-strand cDNA synthesis, but was otherwise treated identically.

### Probes and PCR primers

The PCR primers used to generate probes C, I, L, and M have been published previously [14]. The PCR primers spanning the regions described in Figure 6 were:

P_5'_: 5'-CTTCAAAAACTGCTAGAGATATCGCCGG-3' and

5'-GTACTGGTAGTCCTCTGATGTATGGG-3'

P_3'_: 5'-ATGCCCCGTACGCTTATCTA-3' and 5'-TTTGCCTTTCTAGCCCATGA-3'

P_*dh*_: 5'-CAACACCAATACTGACGATGATG-3' and 5'-GCAATAGAACCAGCGGTTTG-3'

Primers for centromeric *dh *repeats have been published previously [33].

### RNA preparation and reference pool for microarrays

Whole-cell RNA was isolated from *S. pombe *cell pellets (~1.6 × 10^8 ^cells) by hot-phenol extraction and purification with RNeasy columns (Qiagen) following a published protocol [59]. Aliquots (10 μg) were made (henceforth referred to as 'sample RNA') and RNA quality was assessed by UV absorbance, by agarose gel electrophoresis to confirm intact rRNA bands, and by northern hybridization to *act1*^+^. A reference pool consisting of RNA from each sample was made, comprising 76% *trt1*^- ^cells and 24% *trt1*^+ ^cells. This pool was divided into 10 μg aliquots (henceforth referred to as 'reference RNA') and used as the reference RNA in all hybridization experiments reported here.

A single large batch of YES medium was made at the start of the study and used to culture all cells analyzed by microarrays to prevent batch-to-batch medium variations that might yield artifactual microarray results.

### Microarray cDNA labeling, hybridization and data acquisition

The procedures performed and the *S. pombe *microarrays used have been described previously [59]. Whole-cell RNA (10 μg) was labeled by directly incorporating either Cy3-dCTP (reference RNA) or Cy5-dCTP (sample RNA) through reverse transcription. The resulting cDNA was hybridized onto DNA microarrays containing spotted PCR products for over 5,269 different genes and genomic elements printed in duplicate on glass slides representing 99.9% of all known and predicted fission yeast genes. Microarrays were scanned using a GenePix 4000B laser scanner (Axon Instruments) and analyzed with GenePix Pro software. Low-quality signals were filtered out, and data were normalized using a customized Perl script (local adjustment of median of ratios to one within running windows of 1,000 spots).

### Data evaluation and gene classification

Normalized data (Cy5/Cy3 ratios) were evaluated using GeneSpring (Silicon Genetics). All gene-expression values were normalized to the average of two *trt1*^+ ^biological replicates (strains WT 3 and WT 5) collected on day 1 of the growth curve. Experiments and genes were clustered in GeneSpring using the Pearson correlation around zero (termed the Standard correlation in GeneSpring) with a minimum distance of 0.001 and a separation ratio of 1. Gene annotations were taken from GeneDB at the Wellcome Trust Sanger Institute [60]. Lists of genes whose expression changed in the fission yeast stress response [16] were taken from the authors' website [61]. BLAST searches were performed using the NCBI BLAST server [62].

The density of genes with changed regulation along the chromosome was determined by using a running window of 20 consecutive genes along each chromosome [63]. For each window, the probability of obtaining the observed results by chance was calculated using the hypergeometric distribution.

There were two microarray signals - SPAC212.06 (a pseudogene) and the reverse transcript of centromeric *dh *repeats - that we believe were due to cross-hybridization with the SPAC212.11 transcript (or transcripts from identical ORFs, see text). Cross-hybridization becomes apparent with array element sequence identities higher than about 70% [59]. The SPAC212.11 transcript is capable of hybridizing to the entire SPAC212.06 microarray probe (99% sequence identity), but the SPAC212.06 transcript does not contain the sequence required to hybridize to the SPAC212.11 microarray probe. The SPAC212.11 transcript also has a high degree of homology with the *dh *repeat microarray probe sequence (83% sequence identity), and both the SPAC212.11 and *dh *repeat transcripts are expected to be capable of hybridizing to each other's microarray probes. Significant levels of forward and reverse centromeric *dh *repeat transcripts could not be detected using RT-PCR with RNA from days 1, 7, 9 and 15 of the growth curve (Figure 7a) (indicating they could not hybridize to the microarray), although helicase RNA was detected by both RT-PCR and northern hybridization (Figure 7a and data not shown).

Twenty-one microarrays were used in this study, representing two wild-type biological repeats, 15 days of the growth curve, and four strains with circularized chromosomes. The complete raw and normalized data sets are available from ArrayExpress [64] (Accession number: E-MEXP-201).

## Figures and Tables

**Table 1:** Genes with significantly altered expression in *trt1*^- ^cells

Category	Examples	GC	Circ
Acetyltransferase (2)	*ppr1*^+^, SPBC1271.07c*	2-0	1-0
Alcohol metabolism (2)	SPCC24B10.20*, SPAPB24D3.08c*	2-0	0-0
Amino acid and derivative metabolism (6)	SPBC119.03*, SPBPB21E7.04c*, SPAC139.05*	5-1	3-1
Carbohydrate metabolism (14)	*eno102*^+^, *tms1*^+^, *fbp1*^+^, SPCC663.08c*	13-1	8-0
Cell organization (3)	*eng1*^+^, SPBC8E4.10c*, SPAC11D3.01c*	2-1	0-0
Cofactor metabolism (2)	SPAC513.07*, SPAC2E1P3.04* ^§^	1-1	0-0
DNA maintenance and recombination (3)	SPAC212.11*, *trt1*^+^	2-1	0-1
Energy production (5)	SPBC23G7.10c*, SPAC513.02*, SPBC1773.06c*	5-0	2-0
Ion homeostasis (2)	*zym1*^+^, SPBC947.05c*	2-0	1-0
Meiosis and sporulation^‡^(5)	*mfm2*^+†^, *meu3RC*^+^*^†^, *meu8*^+^*^†^, *meu27*^+^, SPBC354.08c*^†^	4-1	0-0
Methyltransferase (1)	SPAC1B3.06c*	1-0	0-0
Mitochondrial energy and proteins (10)	*cox1*^+^, *cox3*^+^, *cob*^+^, *atp6*^+^, *atp8*^+^, *atp9*^+^	10-0	0-0
Nucleotide metabolism (2)	SPBC1683.06c*, SPCC965.14c*	1-0	1-0
Proteolysis (6)	*isp6*^+^, SPBC1685.05*, SPCC338.12*	6-0	1-0
Pseudogene (7)	SPBC16E9.16c*, SPBPB21E7.08*	7-0	3-0
RNA binding and regulation (3)	SPCC74.09*, SPAC4G8.03c*	3-0	1-0
Non-coding RNA (1)	meu3RC	1-0	0-0
Signal transduction (2)	*hri1*^+^, SPBC725.06c*	2-0	1-0
Stress response (8)	*hsp16*^+^, *cta1*^+^, *hsp9*^+^, *ish1*^+^, *pyp2*^+^	8-0	3-0
Sulfur metabolism (2)	*gst2*^+^, SPBC1198.01*	1-0	2-0
Transcription (3)	*aes1*^+^, SPAC30.02c*, SPBC1105.14*	3-0	1-0
Transporter (6)	*cta3*^+^, SPCC1840.12*	5-1	2-0
Unknown function/hypothetical protein (29)	SPAC25H1.01c*	29-0	24-0

The total number of genes in each category is indicated in parenthesis. For each category, the number listed before the hyphen is the number of genes with at least two instances of upregulated expression, and the number after the hyphen is the number of genes with at least two cases of downregulated expression. GC, growth curve; Circ, strains C1 and C5, where numbers represent changes that occurred in both strains. *Putative function. ^†^Meiosis-associated genes with changed expression in the CESR [16]. ^‡^This category contains genes that may also appear in other categories. All other categories are nonredundant. ^§^SPAC2E1P3.04 appears to have been deleted from the genome in all strains except WT 3, WT 5 and C1.

**Table 2:** Maintained expression in strains C1 and C5

Gene name	Category	Gene name	Category
SPBC1271.07c	Acetyltransferase*	*aes1*^+^	Transcription
SPBPB21E7.04c	Amino acid/derivative metabolism*	SPCC1840.12	Transporter*
SPBC119.03	Amino acid/derivative metabolism*	*cta3*^+^	Transporter
SPAC139.05	Amino acid metabolism*	SPBP4G3.03	Unknown/hypothetical
SPBC359.02	Amino acid metabolism*	SPBC660.05	Unknown/hypothetical
SPACUNK4.17	Carbohydrate metabolism*	SPAC25H1.01c	Unknown/hypothetical
SPBC24C6.09c	Carbohydrate metabolism*	SPAC29A4.12c	Unknown/hypothetical
SPAC3G9.11c	Carbohydrate metabolism*	SPBC19C7.04c	Unknown/hypothetical
SPAC4H3.03c	Carbohydrate metabolism*	SPAC15E1.02c	Unknown/hypothetical
SPCC1739.08c	Carbohydrate metabolism*	SPBC1348.03	Unknown/hypothetical
SPCC663.08c	Carbohydrate metabolism*	SPAC23C11.06c	Unknown/hypothetical
SPAC513.02	Carbohydrate metabolism*	SPAC637.03	Unknown/hypothetical
SPCC663.06c	Carbohydrate metabolism*	SPCC584.16c	Unknown/hypothetical
*tms1*^+^	Carbohydrate metabolism	SPBC21C3.19	Unknown/hypothetical
*trt1*^+^	DNA maintenance	SPBC56F2.06	Unknown/hypothetical
SPAC19G12.09	Energy*	SPCC16A11.15c	Unknown/hypothetical
*zym1*^+^	Ion homeostasis	SPCC338.18	Unknown/hypothetical
SPCC338.12	Protease inhibitor*	SPAPB24D3.07c	Unknown/hypothetical
SPBC16E9.16c	Pseudogene	SPCC70.04c	Unknown/hypothetical
SPCC18B5.02c	Pseudogene	SPCC757.03c	Unknown/hypothetical
SPBPB21E7.08	Pseudogene	SPBC1271.08c	Unknown/hypothetical
SPCC70.08c	rRNA methyltransferase*	SPCC191.01	Unknown/hypothetical
SPBC725.06c	Signal transduction*	SPAC27D7.10c	Unknown/hypothetical
*hsp16*^+^	Stress response	SPBC725.10	Unknown/hypothetical
*cta1*^+^	Stress response	SPCC737.04	Unknown/hypothetical
*gst2*^+^	Stress (sulfur metabolism)	SPAC27D7.09c	Unknown/hypothetical
SPAC4H3.08	Stress response (lipid metabolism)*	SPBC725.03	Unknown/hypothetical

Fifty-four genes with maintained expression changes twofold or more in both of strains C1 and C5 that also had changed expression of twofold or more during 2 or more days in the growth curve. All but three genes (*trt1*^+^, *cta3*^+ ^and SPBC359.02) are without changed expression in survivors with linear chromosomes (days 12-15 of growth curve). *Putative function.
